# Prediction of Atrial Fibrillation Recurrence after Thoracoscopic Surgical Ablation Using Machine Learning Techniques

**DOI:** 10.3390/diagnostics11101787

**Published:** 2021-09-28

**Authors:** Sarah W. E. Baalman, Ricardo R. Lopes, Lucas A. Ramos, Jolien Neefs, Antoine H. G. Driessen, WimJan P. van Boven, Bas A. J. M. de Mol, Henk A. Marquering, Joris R. de Groot

**Affiliations:** 1 Heart Centre, Amsterdam UMC, University of Amsterdam, 1105 AZ Amsterdam, The Netherlands; s.w.baalman@amsterdamumc.nl (S.W.E.B.); j.neefs@amsterdamumc.nl (J.N.); a.h.driessen@amsterdamumc.nl (A.H.G.D.); w.j.vanboven@amsterdamumc.nl (W.P.v.B.); b.a.demol@amsterdamumc.nl (B.A.J.M.d.M.); 2 Department of Biomedical Engineering and Physics, Amsterdam UMC, University of Amsterdam, 1105 AZ Amsterdam, The Netherlands; r.riccilopes@amsterdamumc.nl (R.R.L.); l.a.ramos@amsterdamumc.nl (L.A.R.); h.a.marquering@amsterdamumc.nl (H.A.M.); 3 Department of Radiology and Nuclear Medicine, Amsterdam UMC, University of Amsterdam, 1105 AZ Amsterdam, The Netherlands; 4 Department of Clinical Epidemiology and Biostatistics, Amsterdam UMC, University of Amsterdam, 1105 AZ Amsterdam, The Netherlands

**Keywords:** atrial fibrillation, surgical ablation, machine learning

## Abstract

Thoracoscopic surgical ablation (SA) for atrial fibrillation (AF) has shown to be an effective treatment to restore sinus rhythm in patients with advanced AF. Identifying patients who will not benefit from this procedure would be valuable to improve personalized AF therapy. Machine learning (ML) techniques may assist in the improvement of clinical prediction models for patient selection. The aim of this study is to investigate how available baseline characteristics predict AF recurrence after SA using ML techniques. One-hundred-sixty clinical baseline variables were collected from 446 AF patients undergoing SA in our tertiary referral center. Multiple ML models were trained on five outcome measurements, including either all or a number of key variables selected by using the least absolute shrinkage and selection operator (LASSO). There was no difference in model performance between different ML techniques or outcome measurements. Variable selection significantly improved model performance (AUC: 0.73, 95% CI: 0.68–0.77). Subgroup analysis showed a higher model performance in younger patients (<55 years, AUC: 0.82 vs. >55 years, AUC 0.66). Recurrences of AF after SA can be predicted best when using a selection of baseline characteristics, particularly in young patients.

## 1. Introduction

In patients with advanced atrial fibrillation (AF), thoracoscopic surgical ablation (SA) is effective to restore sinus rhythm (SR) [1]. Minimally invasive SA for AF using video-assisted thoracoscopic surgery has increasingly been performed and has a success rate of 69–80% in terms of freedom of AF at one year after surgery [2].

Several clinical variables predicting AF recurrence after catheter ablation (CA) have been identified. These variables are currently being applied for patient selection for both CA and SA [3]. Despite our knowledge of risk factors that are associated with lower efficacy and more recurrences, there are no risk scores or prediction models available that consider all the available pre-procedural clinical data that may affect the outcome of SA. More importantly, it is unknown to what extent the AF recurrence risk after an SA procedure is embedded in baseline clinical characteristics, and to what extent the AF recurrence risk is purely stochastic or related to technical aspects of the procedure (i.e., reconnection across ablation lines). Therefore, a systematic analysis tool to assess the risk of any ablation failure could potentially lead to enhanced identification of patients who may benefit from SA versus those in whom SA therapy would be futile.

Conventionally developed clinical prediction models are using traditional linear regression methods. As an alternative, other machine learning (ML) techniques enable the discovery of (novel) potentially complex patterns in data sets through automated algorithms, using techniques like the kernel trick or multilayer neural network, which may result in more efficient processing of non-linear relationships and complex interactions between variables [4]. ML has already been successfully used on many studies enabling the detection and diagnosis of AF [5]. By using a ML approach, more effective selection and weighing of parameters of choice can be achieved, leading to promising clinical prediction models, which may be more accurate than classical prediction models [6,7]. Still, the ultimate predictive value of such models will depend on the proportion of risk factors present in the variables that are causally related to an outcome versus non-predictive risk factors that are randomly distributed among subjects. Therefore, we sought to optimize the prediction of AF recurrence following SA with the use of available clinical, laboratory and imaging data to investigate to what extent the risk of AF recurrence is already embedded in the preoperative data.

In this study, we built several ML models that incorporate preoperative data in AF patients scheduled for SA to comprehensively predict the AF recurrence risk. The aim of this study was (I) to evaluate the proportion of baseline characteristics that are causal risk factors for AF recurrence after SA using different ML techniques; (II) to investigate the differential performance of ML models on multiple conventional and modified definitions of AF recurrence; and (III) to analyze whether the accuracy of the ML models is pertinent for clinically relevant subgroups.

## 2. Materials and Methods

### 2.1. Patient Characteristics 

Patients with paroxysmal or persistent AF who underwent SA in our center between February 2008 to June 2017 were eligible for this analysis. All patients provided written informed consent before the procedure. Clinical variables collected prior to SA were used for further analysis and consisted of patients’ characteristics, AF type and duration, medical history, the (determinants of the) CHA_2_DS_2_-VASc score, medication, Holter and electrocardiogram (ECG) reports, vital parameters, imaging (i.e., echocardiography, magnetic resonance imaging, computer tomography), and laboratory measurements. A full list of all collected variables is shown in Supplementary Table S1. All continuous variables were standardized by removing the mean and scaling to unit variance. For categorical variables we used one-hot encoding (also known as “dummy coding”).

### 2.2. Procedure and Outcome

Included patients underwent SA following our standard protocol, using a hybrid surgical–electrophysiological approach as described previously [8,9]. Approximately half of the patients underwent additional ganglion plexus (GP) ablation as part of the standard of care in all procedures performed before 2010, or as part of participation in the randomized Atrial Fibrillation Ablation and Autonomic Modulation via Thoracoscopic Surgery (AFACT) trial [2]. As the AFACT trial demonstrated, there was no difference in AF recurrence between the randomized treatment groups, so data of patients with and without GP ablation were pooled. Patients were followed for 24 months after SA with frequent ECG and 24 h-Holter monitoring [2].

Five different definitions of AF recurrence were applied:

- Outcome 1: any episode of atrial tachyarrhythmia (AF, atrial flutter, atrial tachycardia) lasting > 30 s [10].
- Outcome 2: any episode of AF (but not atrial flutter or atrial tachycardia) lasting > 30 s.
- Outcome 3: one single episode of any atrial tachyarrhythmia lasting > 1 h.
- Outcome 4: one single episode of any atrial tachyarrhythmia lasting > 6 h.
- Outcome 5: one single episode of AF (but not atrial flutter or atrial tachycardia), lasting > 1 h.

All outcomes were assessed during the two-year follow-up period, with exclusion of the first three months following the procedure, which were considered a blanking period for outcome analysis. 

### 2.3. Missing Data

Missing data was imputed with MissForest [11], which is an iterative imputation method based on random forest. Only the training set was used to train the imputation model. The target variables (different definition of AF recurrences) were not included in this process. Variables that were less than 70% complete and patients with more than 70% missing data were, sequentially, discarded from the analysis.

### 2.4. Machine Learning Algorithms 

Five well-established ML algorithms were selected: support vector machine (SVM), logistic regression (LR), random forest (RF), neural network (NN), and gradient boosting (GB). All models were implemented using scikit-learn [12]. Furthermore, we applied the least absolute shrinkage and selection operator (LASSO), which performs a regularization to automatically select variables and reduces the number of variables by fitting a linear regression with L1 regularization. This is done to decrease the model’s complexity and reduce the input noise [13]. Variable selection steps are expected to reduce redundant or irrelevant data and can lead to an increase in the model’s accuracy [14].

### 2.5. Analysis Pipeline and Variable Selection

A nested cross-validation (CV), with an internal and external CV, was used for evaluation. The external CV was a stratified 5-fold, which means that 80% of the data was used for training and 20% for testing (repeated five times until all data is used for both training and test). The test set was not used during training and validation steps.

**Figure 1:**
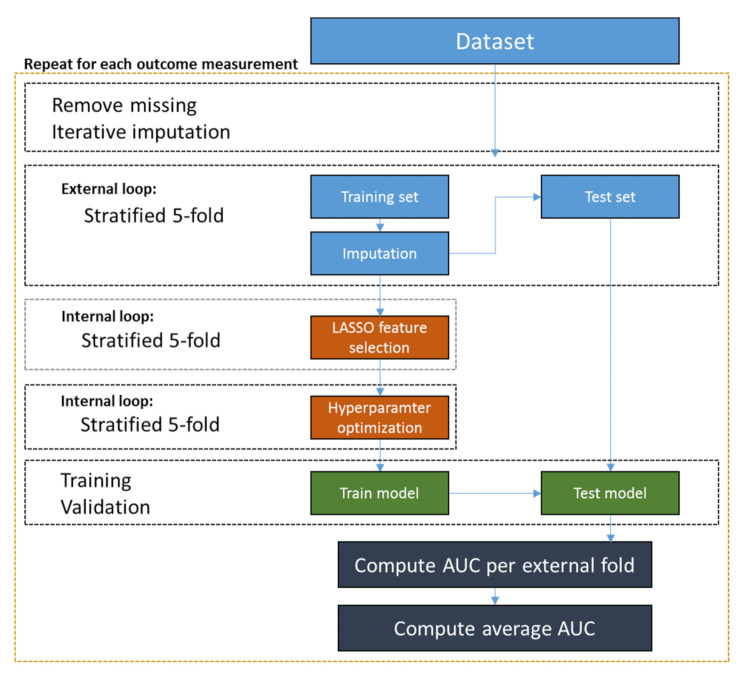
Schematic representation of nested cross-validation methodology. Initially, the missing data is removed and an iterative imputation is performed in a stratified 5-fold CV (external) using only the training set. The imputation model is further used to imput the test set. After that, an internal CV is performed for the LASSO feature selection and hyperparameter optimization. As the last step, the model is trained with the training set and validated with the test set. An average AUC is reported.

The internal CV, also a stratified 5-fold, was first used by LASSO to select the variables to assure that the model generalized well to different data samples. Variables selected more than once in the CV by LASSO were subsequently included to train the models [13]. This strategy was adopted to avoid the chance of selecting a variable that was only meaningful to predict a single fold. Subsequently, the same internal 5-fold CV was used to determine the best hyperparameters by grid search for each classifier on each fold and to train the models. The hyperparameter ranges used are displayed in Supplementary Tables S2 and S3. The pipeline, shown in Figure 1, was ran for all the outcome measurements as target variables.

### 2.6. Model Evaluation

The area under the curve (AUC) of the receiver-operating characteristic was used to evaluate the performance of each model (external CV) and to select the model after the hyperparameter optimization (internal CV). Since a 5-fold CV was used for evaluation, we computed the mean AUC, standard deviation (SD) and confidence interval (CI) of each classifier.

### 2.7. Subgroup Analysis

We performed a predefined subgroup analysis using the model structure (outcome measurement, (key)-variables, ML algorithm) of the two best performing models. For this analysis, the probability prediction from the test sets (from all 5-folds) were combined, creating a single distribution with a single prediction probability for each sample. Samples were selected from this distribution given their subgroups and an AUC was computed for each subgroup individually. Subgroups were chosen based on their established predictive value for AF recurrence or inclusion in the CHA_2_DS_2_-VASc score [15,16]. Variables with an unbalanced distribution were not taken into account. The following variables were included for subgroup analysis: CHA_2_DS_2_-VASc score, congestive heart failure, history of stroke, history of CA, vascular disease, diabetes, hypertension, left atrial volume index (LAVI), sex, and age. Subgroups were created by using the predefined categories in case of categorical variables, and quartiles in case of continuous variables.

### 2.8. Model Interpretation 

To increase the interpretability of our results, we explored the predictive impact of the selected features in our two best performing models. To gain more insight, we applied the unified framework Shapley additive explanations (SHAP) for the interpretation of predictions, which can be used for both linear and non-linear models [17]. The SHAP was calculated for each feature comparing the prediction of the model without that feature. In addition, in cases where LR proved to be the best performing model, we used the coefficients of each feature to provide an interpretation of how each individual feature affected the prediction.

### 2.9. Statistical Analysis

Continuous data are presented as mean (SD) or median (range) for normally and non-normally distributed data, respectively. The unpaired T-test and Mann–Whitney U test were used for comparisons of AUCs between two groups. One-way ANOVA and Kruskal–Wallis tests were used for comparisons of AUCs between more than two groups. Statistical analyses were performed using SPSS Version 26 (IBM Corporation, Armonk, NY, USA). ML were developed with Python programming language 3.6 (Python Software Foundation, Beaverton, OR, United States).

## 3. Results

Of the 495 patients, 49 (10%) patients were excluded because of incomplete baseline data. The mean age of the 446 included patients was 60 (SD ± 9) years, 335 (75%) were male and 266 (60%) had persistent AF (Table 1). An overview of baseline characteristics stratified by success or failure according to different outcome definitions is shown in Supplementary Table S4. In total, 18 out of 160 baseline variables (11%) were excluded because of missing values in more than 30% of the patients.

### 3.1. Prediction of AF Recurrence within Two Years after SA

#### 3.1.1. Outcome 1

**Figure 2:**
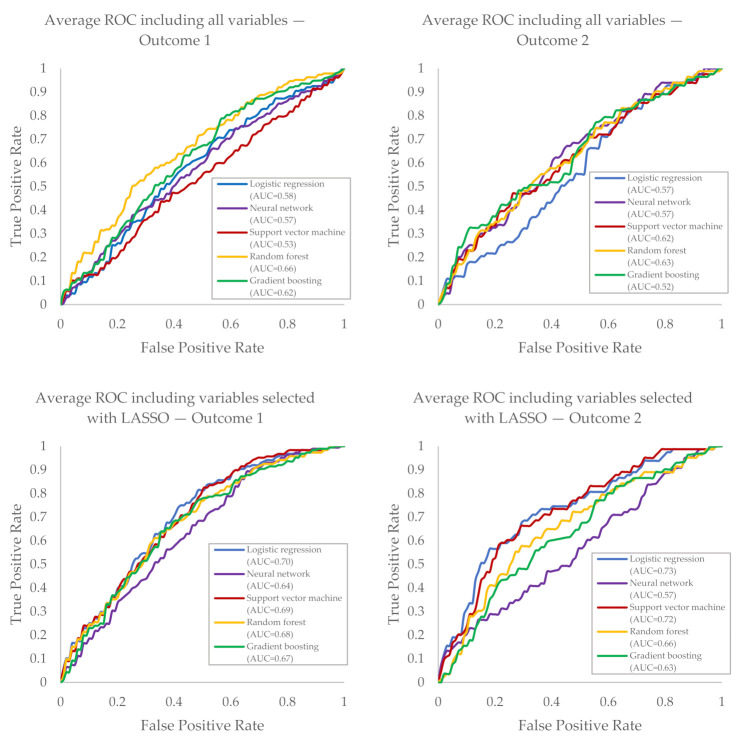
Average 5-fold ROC of testing on Outcome 1 or Outcome 2 with all or a selection of variables for two years without AF recurrence. *ROC* receiver operating characteristic curves, *AUC* area under the curve.

A total of 188 (42%) of the 446 patients experienced recurrence of AF within two years after SA according to the definition of AF recurrence following current guidelines (Outcome 1). Prediction of AF recurrence, and all baseline characteristics, resulted in an AUC varying from 0.53 (95% CI: 0.38–0.68 [SVM]) to 0.66 (95% CI: 0.59–0.72 [RF]) (Figure 2, Table 2). Variable selection using LASSO resulted in a selection of 12 key variables on the 5-fold CV. Variables regarding left atrial (LA) size, age and comorbidity (i.e., use of ACE inhibitors) demonstrated to be the most frequently (100%) selected variables to predict AF recurrence defined as Table 3. Training the models on Outcome 1 with the 12 selected key-variables resulted in an improved AUC up to 0.70 (95% CI: 0.62–0.78 [LR]).

#### 3.1.2. Outcomes 2–5 

In line with the results of Outcome 1, model performance significantly improved for all other outcome definitions using selected key variables instead of using all 142 available variables (*p* < 0.001). There were no significant differences in model performance between all outcome definitions (*p* = 0.35), nor in model performance between different ML techniques (*p* = 0.28). However, the best performing model for Outcome 2 (LASSO, LR) had a higher AUC (0.73, 95% CI: 0.68–0.77) compared to the best performing model of Outcome 1 (LASSO, LR; AUC: 0.70, 95% CI: 0.62–0.78). Figure 2 shows the average 5-fold ROC of model training for Outcome 1 and Outcome 2 with all and a selection of variables.

### 3.2. Variable Selection 

Table 3 shows variable selection by LASSO for the prediction of AF recurrence for the two outcome definitions with the highest model performance (Outcome 1 vs. Outcome 2). In contrast with the selected key variables for Outcome 1, variables regarding comorbidities, but not regarding age or LA size, were the most frequent (100%) selected variables to predict AF recurrence defined as Outcome 2. 

### 3.3. Model Interpretation Analysis 

**Figure 3:**
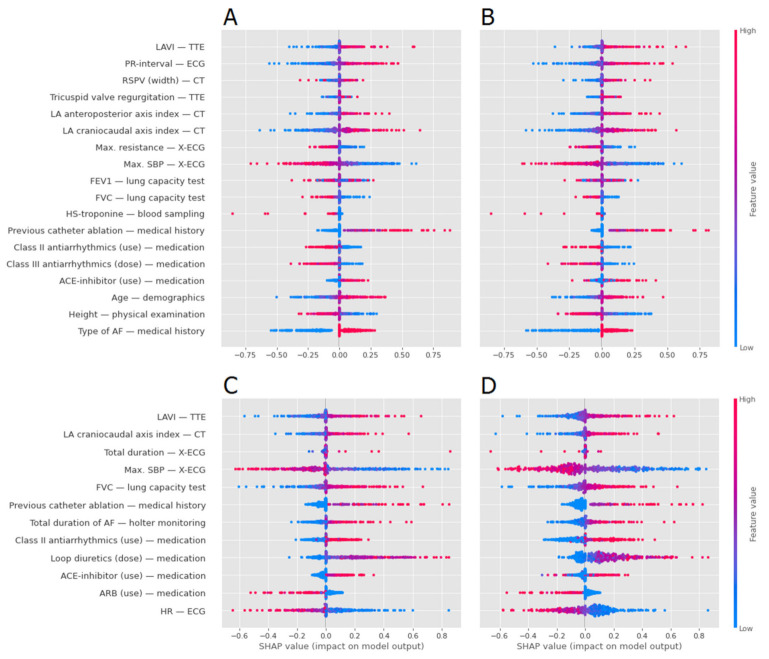
Average feature importance using SHAP values over the test folds. The amplitude of the SHAP value indicates the importance of the feature for the prediction (negative values means good outcome, positive values means bad outcome). The colours represent the value of the features, with red for high values (or true for binary) and blue for low values (or false for binary). The importance was calculated for Outcome 1 (**A**,**B**) and Outcome 2 (**C**,**D**) for the best performing models: LR (**A**,**C**) and SVM (**B**,**D**).

Feature importance (SHAP) of each key variable for the two best prediction models (LR, SVM) regarding Outcome 1 and Outcome 2 was calculated and averaged over the test folds (Figure 3). For both outcomes, the key variables with the highest SHAP values (amplitude) were consistent for the two models. For Outcome 1, AF type, maximal systolic blood pressure (SBP) during exercise testing, increased craniocaudal index of the LA on CT, and PR interval on the baseline ECG were the key variables with the highest SHAP values. Hence, patients with persistent AF had a higher risk of AF recurrence (defined as Outcome 1) than patients with paroxysmal AF. In addition, for the continuous variables, the progressive change in color in Figure 3 indicates a possible linear relationship between the value of the variable and Outcome 1. Patients with a low maximal SBP during exercise testing, increased craniocaudal index of the LA and prolonged PR interval had a higher risk of AF recurrence (Outcome 1). For Outcome 2, maximal SBP during exercise testing, loop diuretics dose and heart rate on the baseline ECG were key variables with the highest SHAP values for both models. There was no difference in the direction of the SHAP values between the models of Outcome 1 and Outcome 2. As LR proved to be the best performing ML technique for both Outcome 1 and Outcome 2, we calculated the average LR coefficients (Supplementary Table S5).

### 3.4. Analysis of AF Recurrence Prediction in Subgroups

**Figure 4:**
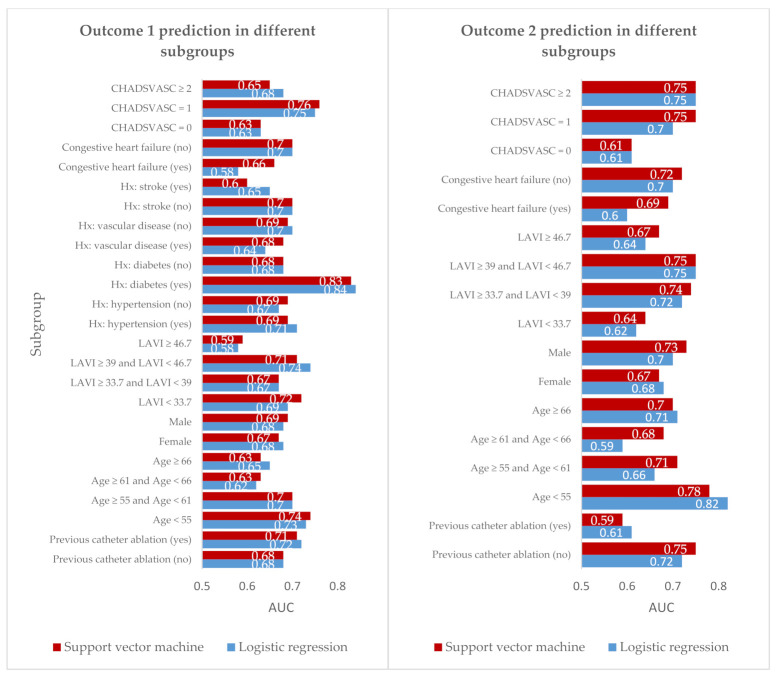
Subgroup analysis with pre-defined groups based on Outcome 1 and Outcome 2 best prediction models.

Figure 4 shows the results of the balanced subgroups ranked by AUC for Outcome 1 and Outcome 2. There was an interaction between model performance and age, with the best performance of the model in patients < 55 years old (AUC: 0.82) for Outcome 2.

## 4. Discussion

This study of 446 patients undergoing SA for paroxysmal or persistent AF in our center aimed to improve patient selection for SA by investigating the value of baseline characteristics for the prediction of AF recurrence. Our main findings are: (I) investigated ML models perform moderately well in the prediction of AF recurrence when all available baseline variables are included, but, with a selection of key variables, the prediction of AF recurrence improves; (II) there are no differences in model performance using modified definitions of AF recurrence or different ML techniques; and (III) subgroup analysis shows an improved model performance in younger patients. 

### 4.1. Prediction of AF Recurrence after Thoracoscopic Surgery

In line with risk scores and predictors for AF recurrence after CA for AF, clinical variables available before SA may predict which patients will benefit from SA. In this study, the use of all available baseline characteristics resulted in a moderate AUC to predict AF recurrence. However, an increased model performance was observed when using a selection of variables. A possible explanation is that input of a selection of key variables leads to less noise and redundancy. The key variables selected by LASSO to predict AF recurrence included LA size, which is a well-known predictor for AF recurrence after AF catheter ablation. Other included key variables were relatively uncommon as stand-alone predictors for AF recurrence. However, these may have been selected because they reflect patients’ levels of frailty and comorbidities which may affect the risk of AF recurrence, or as a reflection (e.g., length) of well-known predictors (e.g., sex) that were not chosen. Surprisingly, patients with a low maximal SBP during exercise testing demonstrated to be at increased risk for AF recurrence. Possibly, this is because this group consists of the foremost advanced AF patients with a higher risk of AF recurrence, who are therefore more aggressively treated with antihypertensive or class II antiarrhythmic medication, or of patients with concomitant diastolic dysfunction. The selected key variables also explain why the model performs better in younger patients. As this patient group consists of patients with fewer comorbidities, it may represent a more homogeneous group with respect to the arrhythmogenic substrate for AF than older patients with multiple comorbidities. 

### 4.2. AF Recurrence Definition and Measurement

Following current guidelines, AF recurrence was defined as any episode of atrial tachyarrhythmia lasting > 30 s beyond the three months blanking period [9]. However, this definition is debatable, as one brief single episode does not carry the same symptom burden as episodes that last days to weeks [18]. Our results did not show any difference in model performance when adjusting the definitions of AF recurrence. The models had a trend towards a higher AUC for Outcome 2 than for Outcome 1. A possible explanation is that recurrent AF may represent an advanced atrial substrate, or progressive disease, whereas recurrent atrial tachycardias may also result from technical failure of the procedure (i.e., reconnection across ablation lines) [19,20]. However, due to the generally low burden of AF recurrence [21], repeat ablation was not performed in a large proportion of these patients and reconnection across ablation lines was not proven. 

### 4.3. Additional Value of ML Techniques in the Prediction of AF Recurrence

It is expected that the application of ML techniques will improve future risk scores and prediction models. Our study shows a very moderate predictive value when using ML models including all available clinical variables as data input. However, using additional techniques, such as LASSO and SHAP, revealed some interesting findings that may improve prediction of AF recurrences after thoracoscopic AF surgery. Our findings underscore that ML tools, particularly those for selection and weighing of variables of interest, may contribute to improvement of prediction models and risk scores. This may be particularly relevant for large data sets with multiple variables wherein regular statistical methods show insufficient correlations. 

### 4.4. Clinical Implications

Improved patient selection for SA could result in a higher success rate of the procedure. In patients with a predicted high risk of AF recurrence, it could be decided not to perform the procedure to prevent the associated complications. In addition, patient selection could identify patients at high risk for AF recurrence that could benefit from additional (continuous) monitoring, other specific follow-up management, and early re-intervention in case of (a)symptomatic AF recurrence. The selection of patients for SA is already based on a thorough preoperative screening based on the patient’s medical history and baseline characteristics. Therefore, the included patients are already part of a highly selected population. This reduces the odds of improving patient selection with the available baseline variables, regardless of the use of ML techniques. As the AF field is evolving, future use of complex in-depth patient characteristics, procedural and mapping data, and improvements of the surgery technology, combined with different feature selection techniques, may further increase model performance.

### 4.5. Limitations

This study has some limitations. First, we only used data from a single center in our test and validation sets. Thereby, it is unknown how our models will perform in other comparable datasets. Furthermore, patients included in this analysis were patients who underwent SA. Patients that did not consent or were deemed unsuitable for the operation were therefore excluded from this analysis. This may impact on the generalizability of our findings. In addition, we did not perform a prospective validation of our models.

AF recurrence was monitored by repetitive ECGs and Holter monitoring as recommended by the guidelines [10]. Patients were encouraged to obtain additional rhythm recording when symptomatic, but no continuous monitoring was performed. Therefore, asymptomatic recurrences of AF may have remained undetected. This could have been avoided by using loop recorders, which were not available for our population. However, the main goal of SA is to reduce AF-related symptoms in patients with advanced AF and thereby improve quality of life. Additionally, no specific indexes for adrenergic tone were available or included in this study. Finally, LASSO is, by definition, a linear regression with L1 regularization selecting features based on the linear correlation. As a result, the linear techniques might have been benefited when this feature selection was performed. The use of non-linear techniques (e.g., the feature importance of the RF) for feature selection, or even simpler techniques, might increase the accuracy of the ML techniques that can handle nonlinearities.

## 5. Conclusions

The proportion of risk of AF recurrence after SA embedded in baseline variables is modest. Advanced ML models predict recurrences of AF after SA best when using a selection of baseline characteristics, particularly in young patients.

## Figures and Tables

**Table 1:** Summarized patients’ characteristics for all included patients.

Variable		No. of Patients (%)
*n*		446
Sex, *n* (%)		
	Male	335 (75.1)
	Female	111 (24.9)
BMI, mean (SD)		25.8 (7.5)
Age, mean (SD)		60.0 (8.7)
CHA_2_DS_2_-VASc, *n* (%)		
	0	122 (27.4)
	1	141 (31.6)
	≥2	183 (41.0)
AF type, *n* (%)		
	Paroxysmal	180 (40.4)
	Persistent	266 (59.6)

**Table 2:** Average area under the curve (AUC) and 95% confidence interval (CI). The rows are the outcomes and the variables included for training the models, and the columns are the machine learning algorithms.

Models, AUCs (95% CI)	Logistic Regression	Neural Network	Support Vector Machine	Random Forest	Gradient Boosting
Outcome 1	0.58 (0.54–0.61)	0.57 (0.44–0.71)	0.53 (0.38–0.68)	0.66 (0.59–0.72)	0.62 (0.55–0.68)
Outcome 1 with LASSO	0.70 (0.62–0.78)	0.64 (0.61–0.67)	0.69 (0.66–0.73)	0.68 (0.63–0.73)	0.67 (0.65–0.69)
Outcome 2	0.57 (0.50–0.64)	0.57 (0.50–0.64)	0.62 (0.55–0.70)	0.63 (0.57–0.69)	0.52 (0.48–0.57)
Outcome 2 with LASSO	0.73 (0.68–0.77)	0.57 (0.50–0.64)	0.72 (0.66–0.78)	0.66 (0.55–0.76)	0.63 (0.54–0.72)
Outcome 3	0.54 (0.48–0.60)	0.54 (0.42–0.65)	0.56 (0.47–0.65)	0.67 (0.61–0.72)	0.68 (0.59–0.76)
Outcome 3 with LASSO	0.69 (0.65–0.74)	0.68 (0.62–0.74)	0.67 (0.63–0.71)	0.69 (0.64–0.75)	0.67 (0.56–0.78)
Outcome 4	0.56 (0.48–0.63)	0.61 (0.57–0.64)	0.56 (0.43–0.69)	0.63 (0.55–0.72)	0.64 (0.52–0.75)
Outcome 4 with LASSO	0.68 (0.59–0.77)	0.62 (0.52–0.73)	0.66 (0.58–0.74)	0.67 (0.58–0.76)	0.65 (0.56–0.73)
Outcome 5	0.56 (0.51–0.62)	0.54 (0.37–0.70)	0.55 (0.42–0.67)	0.55 (0.51–0.59)	0.51 (0.43–0.59)
Outcome 5 with LASSO	0.69 (0.60–0.78)	0.55 (0.35–0.75)	0.66 (0.57–0.75)	0.67 (0.61–0.73)	0.63 (0.58–0.68)

**Table 3:** Key-variables for Outcome 1 and Outcome 2, ranked by the percentage the variable was selected during the 5-fold cross validation (1-fold = 20%). Variables selected by LASSO, in at least two folds (40%), were included for training the models. *AF,* atrial fibrillation; *ARB,* angiotensin receptor blockers; *CT,* computed tomography; *ECG,* electrocardiogram; *FEV1,* forced expiratory volume in one second; *FVC,* forced vital capacity; *HR,* heart rate; *HS-troponine,* high sensitive troponine; *LA,* left atrium; *LAVI,* left atrial volume index; *RSPV,* right superior pulmonary vein; *SBP,* systolic blood pressure; *TTE,* transthoracic echocardiography; *X-ECG,* exercise testing.

Outcome 1		Outcome 2	
Variable—*Assessment at Baseline*	Selection	Variable	Selection
LAVI—*TTE*	100%	Max. SBP—*X-ECG*	100%
PR-interval—*ECG*	100%	ACE-inhibitor (use)—*medication*	100%
LA craniocaudal axis index—*CT*	100%	ARB (use)—*medication*	80%
Max. SBP—*X-ECG*	100%	LAVI—*TTE*	60%
ACE-inhibitor (use)—*medication*	100%	Total duration—*X-ECG*	60%
Age—*demographics*	100%	FVC—*lung capacity test*	60%
LA anteroposterior axis index—*CT*	80%	Class II antiarrhythmics (use)—*medication*	60%
Max. resistance—*X-ECG*	80%	Loop diuretics (dose)—*medication*	60%
Previous catheter ablation—*medical history*	80%	HR—*ECG*	60%
RSPV (width)—*CT*	60%	LA craniocaudal axis index—*CT*	40%
FEV1—*lung capacity test*	60%	Previous catheter ablation—*medical history*	40%
Height—*physical examination*	60%	Total duration of AF — *Holter monitoring*	40%
Type of AF—*medical history*	60%		
Tricuspid valve regurgitation—*TTE*	40%		
FVC—*lung capacity test*	40%		
Hs-troponine—*blood sampling*	40%		
Class II antiarrhythmics (use)—*medication*	40%		
Class III antiarrhythmics (dose)—*medication*	40%		

## Data Availability

The data presented in this study are not publicly available due to privacy and ethical restriction.

## Supplementary Materials

The following are available online at https://www.mdpi.com/article/10.3390/diagnostics11101787/s1, Table S1: All variables and percentage of missing values; Table S2: Hyperparameters grid used for SVM; Table S3: Hyperparameters grid used for RF, GB, and NN; Table S4: Summarized patients characteristics for all included patients divided by outcome definition; Table S5: Average LR coefficients over folds (positive values indicate bad outcomes, and negative values indicate good outcomes).
