# Supplementary material for: Prediction of Atrial Fibrillation Recurrence after Thoracoscopic Surgical Ablation Using Machine Learning Techniques
Source: Diagnostics (Basel). 2021 Sep 28;11(10):1787. doi: 10.3390/diagnostics11101787 (PMC8534896; doi:10.3390/diagnostics11101787)
Supplement: Supplementary file 1 [file diagnostics-11-01787-s001.zip › diagnostics-1355503-supplementary.pdf]

Supplementary Materials

Table S1: All variables and percentage of missing values.

Variable	Missing (%)	Variable	Missing (%)
AF duration (last episode) - *holter monitoring*	81	LIPV (height) - *CT*	12
AF duration (total) - *holter monitoring*	1	LIPV (width) - *CT*	69
Total ECV – *medical history*	24	LSPV (height) - *CT*	7
EHRA score – *symptom score*	33	LSPV (width) - *CT*	66
TV (diameter) - *TTE*	77	PV stenosis - *CT*	12
LAVI - *TTE*	37	RIPV (height) - *CT*	8
Creatinine - *blood sampling*	1	RIPV (width) - *CT*	69
CRP - *blood sampling*	19	RSPV (height) - *CT*	8
eGFR - *blood sampling*	4	RSPV (width) - *CT*	69
Hemoglobin - *blood sampling*	1	TV (diameter) - *CT*	10
INR- *blood sampling*	28	ACE-inhibitor (use) - *medication*	2
Potassium - *blood sampling*	0	ACE-inhibitor (dose) - *medication*	65
Leukocytes - *blood sampling*	1	ARB (use) - *medication*	2
Sodium - *blood sampling*	0	ARB (dose) - *medication*	70
NT-proBNP - *blood sampling*	7	Calcium antagonist (use) - *medication*	2
Thromobytes - *blood sampling*	1	Calcium antagonist (dose) - *medication*	78
Hs-troponine - *blood sampling*	42	Lipid lowering drugs (use) - *medication*	2
TSH - *blood sampling*	7	Lipid lowering drugs (dose) - *medication*	65
Urea - *blood sampling*	37	Class IA antiarrhythmics (use) - *medication*	3
Pulmonary FEV (absolute) - *lung capacity test*	14	Class IA antiarrhythmics (dose) - *medication*	84
Pulmonary FEV (relative) - *lung capacity test*	14	Class IC antiarrhythmics (use) - *medication*	2
Pulmonary FEV1VC (absolute) - *lung capacity test*	14	Class IC antiarrhythmics (dose) - *medication*	60
Pulmonary FEV1VC (relative) - *lung capacity test*	14	Class II antiarrhythmics (use) - *medication*	1
Pulmonary FVC (absolute) - *lung capacity test*	14	Class II antiarrhythmics (dose) - *medication*	44
Pulmonary FVC (relative) - *lung capacity test*	14	Class III antiarrhythmics (use) - *medication*	1

Arrhythmia - *X-ECG*	8
Duration - *X-ECG*	7
Ending - *X-ECG*	7
Ischemia - *X-ECG*	7
Max. HR - *X-ECG*	6
Max. DBP - *X-ECG*	6
Max. SBP - *X-ECG*	6
METS - *X-ECG*	45
Min. HR - *X-ECG*	7
Min. DBP - *X-ECG*	7
Min. SBP - *X-ECG*	7
WATT - *X-ECG*	57
AVB - *ECG*	6
Axis - *ECG*	6
HR - *ECG*	4
PR interval - *ECG*	24
QRS interval - *ECG*	4
QT interval - *ECG*	5
QTc - *ECG*	5
Rhythm - *ECG*	4
Ventricular conduction - *ECG*	5
Aortic valve regurgitation - *TTE*	40
Aortic valve stenosis - *TTE*	36
Mitral valve regurgitation - *TTE*	26
Mitral valve stenosis - *TTE*	42
Pulmonary valve regurgitation - *TTE*	83
Pulmonary valve stenosis - *TTE*	77
Tricuspid valve regurgitation - *TTE*	37
Tricuspid valve stenosis - *TTE*	52
Type of failure - *holter monitoring*	0

Class III antiarrhythmics (dose) - *medication*	53
Class IV antiarrhythmics (use) - *medication*	2
Class IV antiarrhythmics (dose) - *medication*	75
Other antiarrhythmics (use) - *medication*	2
Other antiarrhythmics (dose) - *medication*	74
Loop diuretics (dose) - *medication*	76
Loop diuretics (use) - *medication*	2
Nitrates (use) - *medication*	3
Nitrates (dose) - *medication*	84
OAC (use) - *medication*	0
OAC (dose) - *medication*	79
Potassium diuretics (use) - *medication*	3
Potassium diuretics (dose) - *medication*	82
Thiazide diuretics (use) - *medication*	2
Thiazide diuretics (dose) - *medication*	76
Antiplatelet drug (use) - *medication*	3
Antiplatelet drug (dose) - *medication*	82
Amiodarone - *medication*	13
Atenolol - *medication*	14
Bisoprolol - *medication*	13
Carvedilol - *medication*	13
Digoxin - *medication*	14
Diltiazem - *medication*	14
Disopyramide - *medication*	14
Flecainide - *medication*	10
Quinidine - *medication*	14
Metoprolol - *medication*	10
Nebivolol - *medication*	14
Propafenone - *medication*	14
Propranolol - *medication*	13

AF - *holter monitoring*	8	Sotalol - *medication*	11
Atrial flutter - *holter monitoring*	8	Verapamil - *medication*	14
Atrial tachycardia - *holter monitoring*	9	Age - *demographics*	0
AV block - *holter monitoring*	9	BMI – *physical examination*	0
Mean HR - *holter monitoring*	10	Height - *physical examination*	0
Max. HR - *holter monitoring*	8	HR - *physical examination*	1
Min. HR - *holter monitoring*	8	HR regular - *physical examination*	8
Flutter ablation - *medical history*	64	SBP - *physical examination*	2
Other cardiac procedure - *medical history*	9	Weight - *physical examination*	0
Cardiac surgery - *medical history*	0	DBP - *physical examination*	2
Catheter ablation, PVI - *medical history*	0	Age ≥ 65 - *demographics*	0
Catheter ablation, other - *medical history*	68	Age ≥ 75 - *demographics*	0
All PV - *Catheter ablation, PVI*	81	Alcohol - *intoxications*	55
Entry block - *Catheter ablation, PVI*	81	$CHADS_2$ - *risk score*	0
Exit block - *Catheter ablation, PVI*	81	CHA_2DS_2-VASc - *risk score*	0
Lesions - *Catheter ablation, PVI*	81	Hypercholesterolemia - *medical history*	20
CHF - *medical history*	9	Congestive heart failure - *medical history*	0
MI - *medical history*	0	Diabetes mellitus - *medical history*	0
Pacemaker - *medical history*	0	Drugs - *intoxications*	37
PCI - *medical history*	0	Family history of CVD - *medical history*	37
Surgical ablation - *medical history*	69	Female - *demographics*	0
Heart valve surgery - *medical history*	9	Hypertension - *medical history*	0
Aberrant PV - *CT*	4	Smoking - *intoxications*	22
LA anteroposterior axis index - *CT*	7	Stroke - *medical history*	0
LA craniocaudal axis index - *CT*	10	Vascular disease - *medical history*	0

Table S2: Hyperparameters grid used for SVM.

Classifier	Kernel type	Penalty parameter *C*	Kernel coefficient γ	Degree of the Polynomial kernel	Class weight
SVM	Linear	[0.1, 1, 10, 100, 1000]	n.a.	n.a.	[None, Balanced]
	Radial basis function	[0.1, 1, 10, 100, 1000]	[1, 0.1, 0.01, 0.001]	n.a.	[None, Balanced]
	Polynomial	[0.1, 1, 10, 100, 1000]	[1, 0.1, 0.01, 0.001]	[3, 4, 5]	[None, Balanced]
	Sigmoid	[0.1, 1, 10, 100, 1000]	[1, 0.1, 0.01, 0.001]	n.a.	[None, Balanced]

Table S3: Hyperparameters grid used for RF, GB, and NN.

Classifier	Parameter name	Parameter value
RF	Number of trees	[100, 500, 1000, 2000]
	Max features	[None, auto]
	Max depth	[None, 2, 3, 4]
	Min samples per split	[2, 4, 8]
	Min samples per leaf	[1, 2, 4]
	Class weight	[None, Balanced]
GB	Number of trees	[10, 50, 100, 200, 500]
	Max features	[None, auto]
	Max depth	[None, 2, 3, 4]
	Min samples per split	[2, 4, 8]
	Min samples per leaf	[1, 2, 4]
	Class weight	[None, Balanced]
NN	Activation	[relu]
	Hidden layer sizes	[5], [10], [50], [5, 5], [10, 10], [50, 50], [5, 5, 5], [10, 10, 10], [50, 50, 50]
	Alpha	[0.001, 0.0001]
	Solver	[adam]
	Learning rate	[adaptive]
	Initial learning rate	[0.1, 0.01, 0.001]

Table S4: Summarized patients characteristics for all included patients divided by outcome definition.

Grouped by outcome	Outcome 1 *(n=446)*		Outcome 2 (n=446)		Outcome 3 (n=446)		Outcome 4 (n=446)		Outcome 5 (n=446)	
	**Success**	**Failure**	**Success**	**Failure**	**Success**	**Failure**	**Success**	**Failure**	**Success**	**Failure**
n	*258*	*188*	*363*	*83*	*270*	*176*	*290*	*156*	*367*	*79*
**Gender, n (%)**										
Male	208 (80.6)	127 (67.6)	274 (75.5)	61 (73.5)	215 (79.6)	120 (68.2)	230 (79.3)	105 (67.3)	277 (75.5)	58 (73.4)
Female	50 (19.4)	61 (32.4)	89 (24.5)	22 (26.5)	55 (20.4)	56 (31.8)	60 (20.7)	51 (32.7)	90 (24.5)	21 (26.6)
**BMI, mean (SD)**	25.8 (7.2)	25.8 (7.8)	25.6 (7.8)	26.9 (5.8)	25.7 (7.2)	26.0 (7.8)	25.6 (7.6)	26.2 (7.3)	25.6 (7.7)	27.0 (5.9)
**Age, mean (SD)**	58.8 (8.7)	61.6 (8.4)	59.9 (8.6)	60.5 (9.1)	59.2 (8.7)	61.3 (8.5)	59.2 (8.9)	61.5 (8.2)	59.9 (8.6)	60.5 (9.3)
**CHA_2DS_2-VASc, n(%)**										
0	85 (32.9)	37 (19.7)	105 (28.9)	17 (20.5)	87 (32.2)	35 (19.9)	92 (31.7)	30 (19.2)	106 (28.9)	16 (20.3)
1	76 (29.5)	65 (34.6)	106 (29.2)	35 (42.2)	8 1 (30.0)	60 (34.1)	89 (30.7)	52 (33.3)	109 (29.7)	32 (40.5)
>=2	97 (37.6)	86 (45.7)	152 (41.9)	31 (37.3)	102 (37.8)	81 (46.0)	109 (37.6)	74 (47.4)	152 (41.4)	31 (39.2)
**AF type**										
Paroxysmal	130 (50.4)	50 (26.6)	154 (42.4)	26 (31.3)	137 (50.7)	43 (24.4)	141 (48.6)	39 (25.0)	157 (42.8)	23 (29.1)
Persistent	128 (49.6)	138 (73.4)	209 (57.6)	57 (68.7)	133 (49.3)	133 (75.6)	149 (51.4)	117 (75.0)	210 (57.2)	56 (70.9)

Table S5: Average LR coefficients over folds. Positive values indicates bad outcome and negative values indicates good outcome.

Outcome 1		Outcome 2	
**Variable**	**Average**	**Variable**	**Average**
LAVI - *TTE*	0.011	LAVI - *TTE*	0.028
PR-interval - *ECG*	0.010	LA craniocaudal axis index - *CT*	0.020
RSPV (width) - *CT*	0.010	Duration - *X-ECG*	0.004
Tricuspid valve regurgitation - *TTE*	0.002	Max. SBP - *X-ECG*	-0.019
LA anteroposterior axis index - *CT*	0.011	FVC - *lung capacity test*	0.022
LA craniocaudal axis index - *CT*	0.036	Previous catheter ablation - *medical history*	0.372
Max. resistance - *X-ECG*	-0.003	AF duration (total) - *holter monitoring*	0.027
Max. SBP - *X-ECG*	-0.013	Class II antiarrhythmics (use) - *medication*	0.103
FEV1 - *lung capacity test*	-0.065	Loop diuretics (dose) - *medication*	0.036
FVC - *lung capacity test*	-0.103	ACE-inhibitor (use) - *medication*	0.054
Hs-troponine - *blood sampling*	-0.019	ARB (use) - *medication*	-0.140
Previous catheter ablation - *medical history*	0.439	HR - *ECG*	-0.019
Class II antiarrhythmics (use) - *medication*	-0.088		
Class III antiarrhythmics (dose) - *medication*	-0.001		
ACE-inhibitor (use) - *medication*	0.028		
Age - *demographics*	0.024		
Height - *physical examination*	-0.679		
Type of AF - *medical history*	0.621		
